# Indulging into the Enigma of the Central Giant Cell Granuloma: A Case Report

**DOI:** 10.7759/cureus.65068

**Published:** 2024-07-21

**Authors:** Shweta Sarangi, Lakshmi Rathan A C, Vivek Narayanan, Prashanthi Gurram, Abinaya Subramanian

**Affiliations:** 1 Department of Oral and Maxillofacial Surgery, Sri Ramaswamy Memorial Kattankulathur Dental College and Hospital, Chengalpattu, IND; 2 Department of Oral and Maxillofacial Surgery, Sri Ramaswamy Memorial Kattankulathur Dental College and Hospital, Sri Ramaswamy Memorial Institute of Science and Technology, Chengalpattu, IND

**Keywords:** radicular cyst, cortical expansion, reparative, jaw lesions, central giant cell granuloma

## Abstract

The oral cavity surprises us with a humongous variety of lesions. Central giant cell granuloma (CGCG) is one such rare presentation. The etiology of CGCG is controversial, which ranges from initially being considered a reparative lesion to currently being hypothesized as a mesenchymal proliferative jaw lesion. Clinically, CGCG is not a typical presenting lesion. It may be asymptomatic or even manifest as a slow-growing swelling. This entity most commonly occurs in younger females, particularly situated in the mandible. Here is a case report of a 31-year-old female with CGCG.

## Introduction

Pathological lesions presenting in the oral cavity are often tricky to diagnose. They can encompass various structures of the oral cavity and are often attributed to a multitude of etiological factors, including infections, trauma, neoplasms, systemic disorders, autoimmune disorders, and so on. These entities require a thorough clinical examination as well as diagnostic investigations for accurate identification and management.

Central giant cell granuloma (CGCG) is a comparatively uncommon bony lesion, which is benign in nature. Approximately 7% of all benign jaw lesions are accounted for by this locally aggressive entity [1,2]. In the craniofacial region, the jaw bones are often affected, often involving the mandible over the maxilla, with a relative ratio of 2:1. It is in the mandible that this lesion usually habitats the body, ideally anterior to the first molar. Since this lesion presents in the tooth-bearing area of the craniofacial region, it could be confused with endodontically originating entities. Females are predominantly affected with a peak age group of 10-25 years [3]. Though histologically giant cells are present, the etiopathogenesis of CGCG is controversial and unclear. It is currently assumed to be a reactive, infective, inflammatory, or neoplastic process following a former trauma.

From asymptomatic, sluggish, and indolent to aggressive and rapid bone resorption, the clinical behavior of CGCG may vary greatly. Other salient features noted are cortical expansion, leading to thinning and perforation, root resorption, and displacement of neighboring structures such as teeth and nerves, all of which are frequently accompanied by pain. Though the lesion is unencapsulated, it does not infiltrate the adjacent structures. About 15% to 20% of recurrence is common, especially in cases of the more aggressive variant [4].

The management of CGCG presents numerous challenges. The first choice of treatment is surgical excision. Nonetheless, there are now medical therapy alternatives to treat recurrent aggressive CGCG.

We hereby present and discuss the case of a 31-year-old female who was first misdiagnosed with a radicular cyst, and approached us with pain in her teeth despite undergoing partial treatment for the same.

## Case presentation

A 31-year-old female patient presented to our Department of Oral and Maxillofacial Surgery, with a chief complaint of pain in the left lower front teeth for 3 months. She had approached a private practitioner around 1 year ago with a similar complaint. A periapical radiolucency was noted in the radiograph of 33 and 34 teeth. Endodontic treatment was initiated for these teeth, owing to the diagnosis of the radicular cyst. Despite undergoing treatment, the symptoms never subsided.

Currently, the patient presented with sensitivity in the left lower premolars for 6 months. Three months later, this was succeeded by pain. The pain was gradual on onset, continuous in nature, throbbing type, aggravated on consumption of food, and relieved on medication. The pain was not associated with any other symptoms like swelling, fever, and so on. The patient did not report any other relevant medical history.

**Figure 1:**
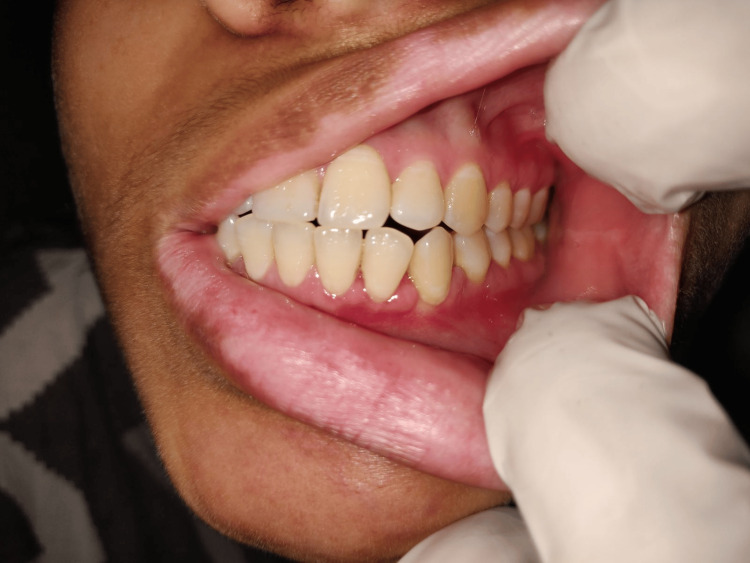
Pre-operative clinical image depicting no obvious clinical signs

Extraoral examination did not reveal any gross facial symmetry. No lymph nodes were also palpable. On intraoral examination, no ulceration or erythema was observed. Access cavity preparation was evident with 33 and 34 teeth (Figure 1). None of the teeth were mobile. On palpation, tenderness is evident in relation to the left buccal vestibule extending from 31 to 35 region.

**Figure 2:**
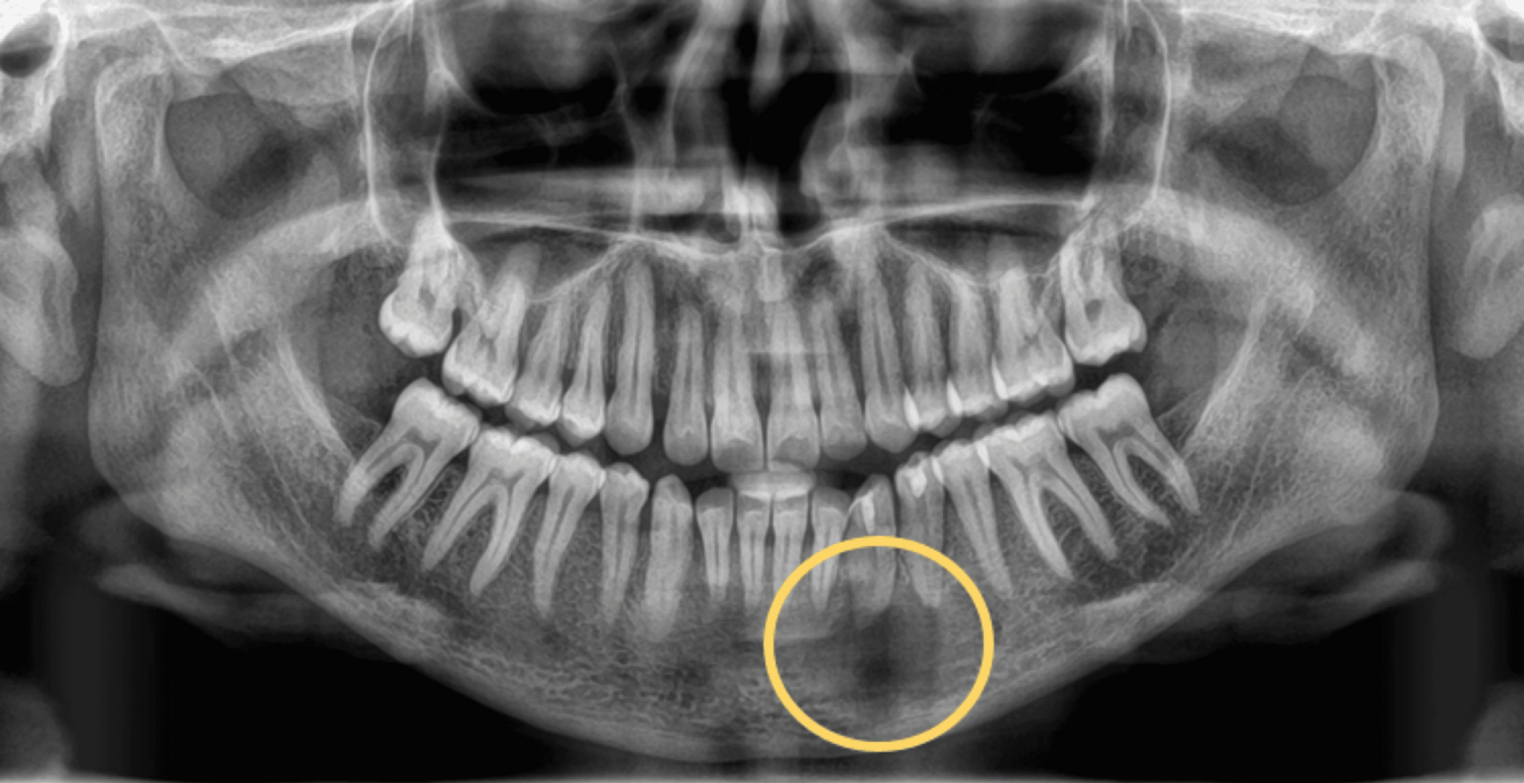
Pre-operative orthopantomogram showing a radiolucency at the periapical region of 32, 33, and 34

**Figure 3:**
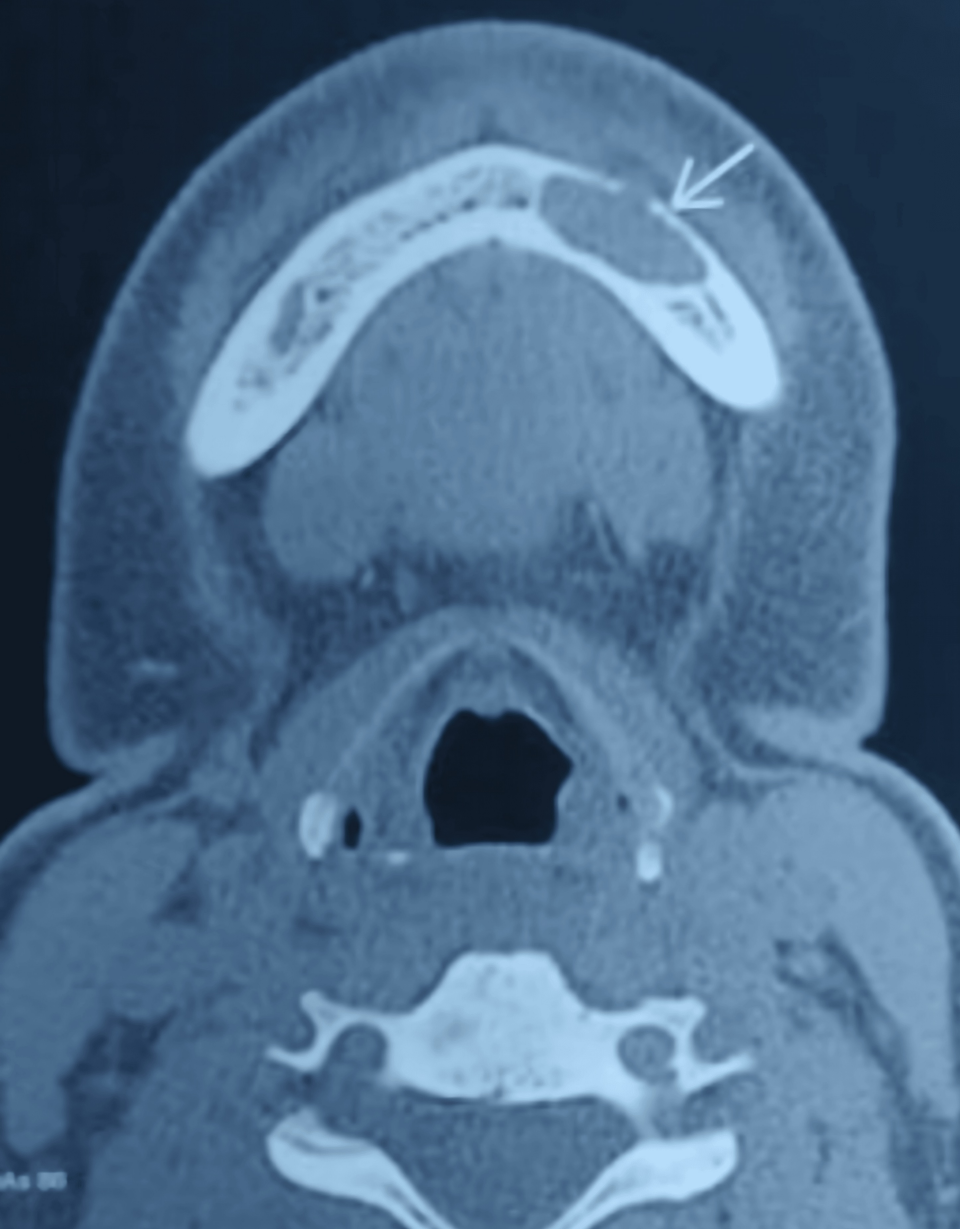
Pre-operative CT image showing the lesion in the left anterior mandible CT: computed tomography

All routine blood and biochemical investigation results were within normal range. A pulp vitality test was conducted for 31, 32, 33, 34, 35, and 36 teeth, which was negative for 32, 33, and 34. Various radiological investigations were conducted. The orthopantomogram revealed an ill-defined, unilocular radiolucency at the apex of 33 and 34 (Figure 2). CT facial bones were also performed on the patient revealing an ill-defined radiolucency measuring about 3×2 cm was present in the left anterior portion of the mandible, ahead of the left mental foramen (Figure 3).

This entity was provisionally diagnosed as a radicular cyst related to 33 and 34. The differential diagnoses considered were odontogenic keratocyst, ameloblastoma, aneurysmal bone cyst, and CGCG.

**Figure 4:**
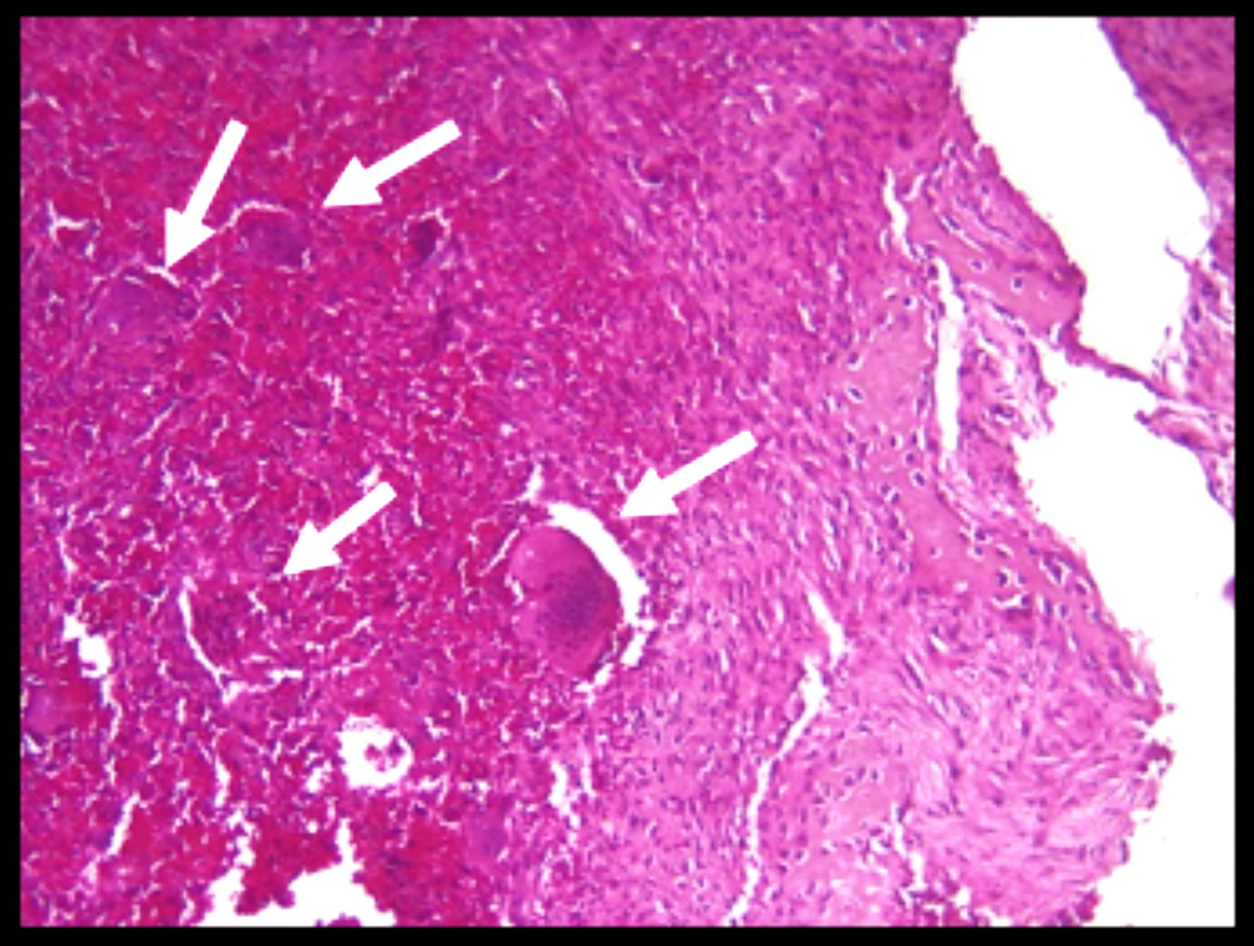
: Histopathology The presence of numerous giant cells of varying size and shapes often found in clusters scattered throughout the lesion in a fibrovascular stroma chiefly of mononuclear cells admixed with areas of hemorrhage.

The patient was subjected to an incisional biopsy, for which histopathological analysis was performed. The histopathological examination displayed fibrovascular connective tissue with increased vascularity. The stroma showed areas of extravasated RBCs, admixed with a chronic type of inflammatory cells, chiefly multinucleated giant cells of varying size and shapes. Individual giant cells of varying sizes with 5-25 nuclei along with numerous normal-looking trabeculae of bone were noted (Figure 4).

The conglomeration of clinical, radiographic, and histologic features paved the way to diagnose the patient with CGCG of the left anterior mandible. Excision of the tumor under general anesthesia was proposed and hence executed.

**Figure 5:**
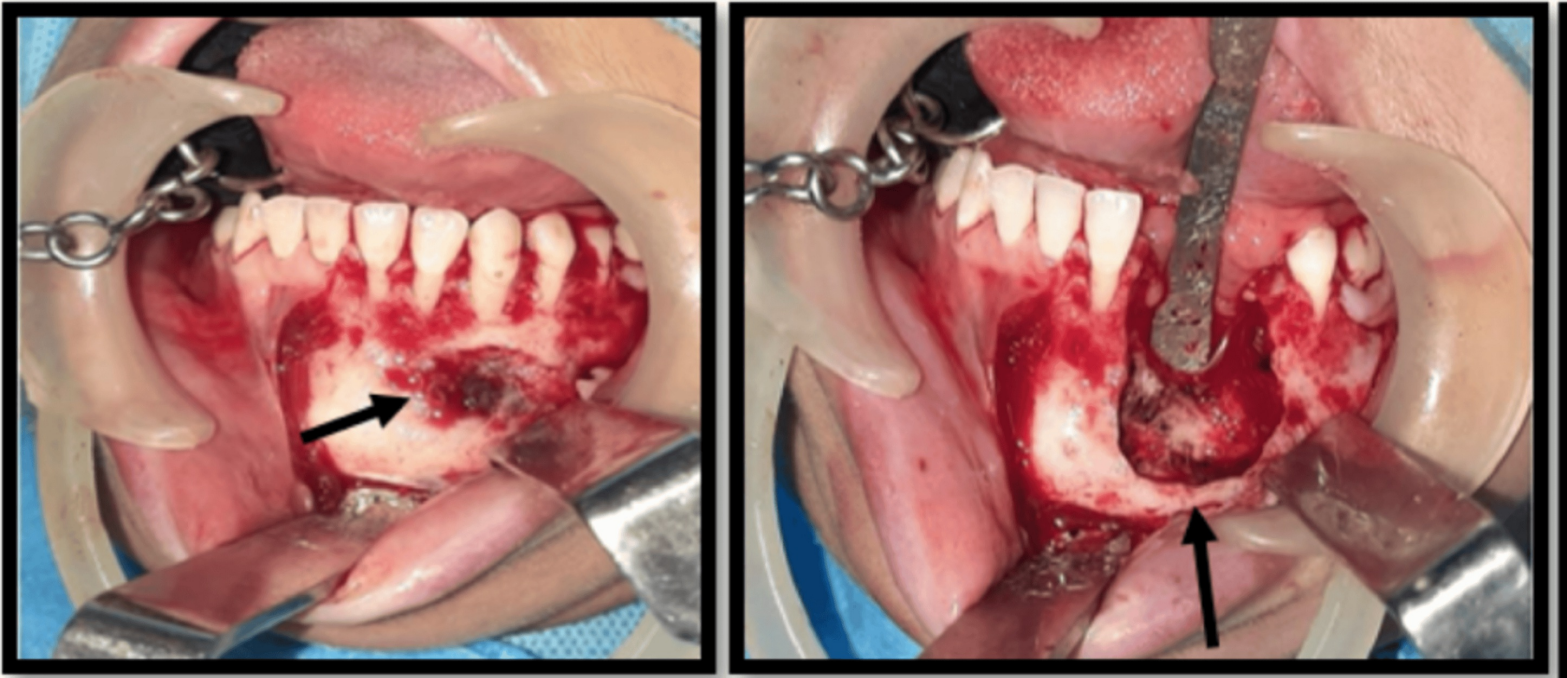
Intraoperative pictures – excision of lesion along with extraction of involved teeth a) Lesion extending from 32, 33, and 34 regions exposed through crevicular approach; b) post-complete enucleation, curettage, and extraction of 32, 33, and 34 teeth

Under right nasoendotracheal intubation, GA was induced and maintained. Standard patient preparation and draping were done. Local anesthesia (2% lignocaine with 1:80,000 adrenaline) was infiltrated in the left lower vestibule. A crevicular incision was placed that extended from 35 to 41, with releasing incisions on either side. The mucoperiosteum was reflected using Molt’s periosteal elevator. The tumor concerning 32, 33, and 34 was visualized and exposed. Using a hard tissue curette, the lesion with its lining was excised. Extraction of 32, 33, and 34 was simultaneously performed. Peripheral osteotomy was done. Interdental papillae were trimmed, and a neat closure was achieved using 3-0 vicryl (Figure 5).

**Figure 6:**
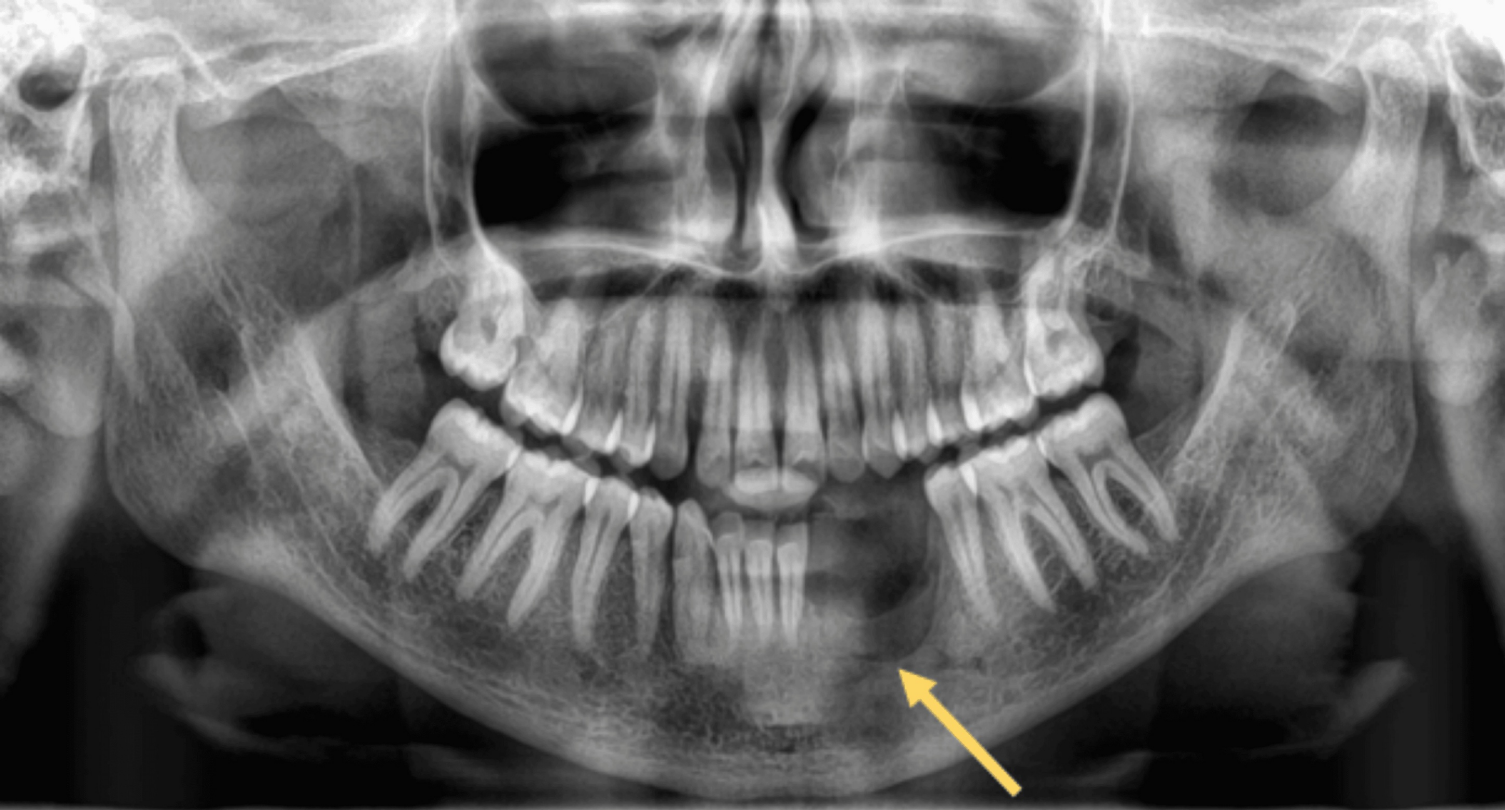
One-year postoperative orthopantomogram shows sufficient bone formation in the defect site in the 32, 33, and 34 teeth region

The patient was periodically monitored up to 1 year after surgical excision of the lesion. No recurrence was observed (Figure 6). The patient was provided with a temporary partial denture postoperatively to maintain function.

## Discussion

Jaffe in 1953 coined the term “giant cell reparative granuloma” as he believed that this lesion is a local, reparative reaction of bone to inflammation, trauma, or hemorrhage. The word “reparative,” though initially used, has been eliminated since clinically, the behavior of these lesions has not been compatible with a reparative process; rather, these lesions must be removed or treated, and they are neither self-limiting nor self-healing [5].

The World Health Organization in 1992 defined CGCG as an intraosseous lesion containing multiple foci of multinucleated giant cells, hemorrhage, and trabeculae of woven bone in fibrous tissue [6].

CGCG can occur in all parts of the body. However, predominantly it is found in the jaws. Characterized by facial swelling and tenderness, clinically CGCG presentation ranges from an asymptomatic, slow-growing swelling to a rapidly expanding aggressive lesion. The high recurrence rate is attributed to the fast-growing variety, particularly when the lesion involves the surrounding soft tissue, surpassing the perforated cortical plates [7,8].

The radiological appearance of CGCG is variable; it may be a unilocular or multilocular radiolucency. Well or ill-defined, it shows expansion as well as destruction of the cortical bone. CGCG does not have any pathognomonic radiographic features, thus confusing it with other radiolucent lesions found in jaws [9-12].

Finally, diagnosing CGCG depends on the histopathology because of the non-specific clinical and radiological features. Histologically, CGCG contains a vascular stroma within which a focal arrangement of giant cells along with adjacent thin-walled capillaries is appreciated. A spindle cell stroma is mostly seen, which perhaps is the cell from which this lesion originates. Similar findings have been observed in our case as well. The key feature that differentiates CGCG from a giant cell tumor is the presence of foreign body-type giant cells.

Although this lesion can be found in all age groups, it is predominantly seen in people below 30 years of age. Commonly detected in the mandible rather than the maxilla, CGCG has a women’s predilection (ratio of female to male is 2:1). Most of the mandibular lesions occur ahead of the first molar and often cross the midline [13-17].

Surgery is the most accepted, established form of treatment for CGCG [8]. However, the extent of excision varies from simple curettage to en-bloc resection. Curettage alone or alongside peripheral ostectomy is the proposed conservative surgical treatment. Laser or cryoprobes may also be used to thermally sterilize the margins of the lesion. Radical surgical approaches, including en-bloc resection, peripheral ostectomy, and resection without continuity defect, have been validated for aggressive CGCG [18].

Some studies have shown positive results in the usage of nonsurgical methods like systemic calcitonin, intralesional injection with corticosteroids, and radiotherapy [12,13]. Over the last few years, the action of inhibiting osteoclastic activity has favored the use of steroids and calcitonin. Conservative management is essential in pediatric patients to prevent long-term developmental defects.

Alpha interferon was proposed because of its action as a mediator to differentiate osteoblasts from mesenchymal cells. Also, its antiangiogenic potential leads to osteogenesis [19]. When aggressive CGCG is administered with alpha interferon (IFN) subcutaneously (3 million units/m^2^ of body surface area) marked improvement is noticed. Stalling of the rapid growth, reduction in size, and consolidation of bone are the few features. However, alpha IFN can only be used as an adjunct. Such aggressive variants need surgical intervention for elimination [20]. The lesions that are treated both pharmacologically and surgically usually show a 71% success rate. Recurrence is rare (5-11%), however, the aggressive variant of CGCG can recur [18].

## Conclusions

The clinical, radiological, biological, and most importantly, anatomopathological nature of CGCG forms the base for its diagnosis. As seen in our case, the lesion presented like it had an endodontic origin and could have been misdiagnosed. The differential diagnoses for CGCG are odontogenic keratocyst, ameloblastoma, brown’s tumor, giant cell tumor, aneurysmal bone cyst, and fibro-osseous lesions like cherubism, to name a few. To rule out all other entities, calcium and phosphorus blood levels must be assessed.

The surgical exploration performed is usually conservative and non-vandalizing in nature, despite the lesion having such a significant volume.

However, block resection is the ideal cure for eradicating this condition. Our aggressive approach, i.e., surgical excision of the lesion was justified. Due to potential relapse, all cases require significant regular monitoring.
